# Dataset of acute oral toxicity and subacute neurotoxicity risk assessments of flavonoid-enriched fraction extracted from *Oroxylum Indicum* on Sprague Dawley rats

**DOI:** 10.1016/j.dib.2023.109411

**Published:** 2023-07-15

**Authors:** Farah Amna Othman, Anani Aila Mat Zin, Yusmazura Zakaria, Nik Nur Hakimah Nik Salleh, Suat Cheng Tan

**Affiliations:** a School of Health Sciences, Health Campus, Universiti Sains Malaysia, Kubang Kerian, 16150, Kelantan, Malaysia; b Pathology Department, School of Medical Sciences, Health Campus, Universiti Sains Malaysia, Kubang Kerian, 16150, Kelantan, Malaysia

**Keywords:** Oroxylum indicum, Flavonoid-Enriched fraction, Acute oral toxicity, Subacute neurotoxicity, Safe dosage

## Abstract

*Oroxylum indicum* is a medicinal herb that garnered enormous attention in drug discovery for human diseases such as neurodegenerative, cardiovascular, arthritis and hepatitis diseases. Pharmacokinetic study confirmed that the pharmacological actions of this herb are associated with its prominent flavonoid bioactive components. Here, the data set of liquid chromatography-mass spectroscopy (LC-MS), neurological functions, relative organ weight (ROW), hematological, biochemical and histopathological parameters of flavonoid-enriched fraction (FEF)-treated Sprague Dawley (SD) rats were presented. The data set was generated from three study groups namely: Sighting Study, Acute Toxicity Study and Subacute Neurotoxicity Study with study duration of 14 days (for Sighting Study and Acute Toxicity Study) and 28 days (for Subacute Neurotoxicity Study) by strictly following the procedures set in Organisation for Economic Co-operation and Development (OECD) Guidelines 420 and 424 in vivo. Rats in sighting study were treated with dosage of 5, 50, 300 and 2000 mg/kg FEF (*n* = 1/dosage/gender), respectively, and were observed for mortality, toxicity signs and behavioural changes. The highest dosage at which none of the animal showed sign of mortality in the sighting study was selected as the test dosage for subsequent acute toxicity study (*n* = 5/dosage/gender). Meanwhile, for subacute neurotoxicity study, SD rats (*n* = 5/dosage/gender) were treated with repeated dosage of 50 mg/kg for 28 days. Neurological behaviours of treated rats were observed daily, while their body weight were measured weekly. Whole blood was collected at the end of the study via cardiac puncture into ethylenediaminetetraacetic acid (EDTA) tubes for hematological evaluation that included the measurements of red blood cells (RBC), hemoglobin (Hb), packed cell volumes (PCV), mean corpuscular volume (MCV), mean corpuscular hemoglobin (MCH), mean corpuscular haemoglobin concentration (MCHC), platelet, white blood cells (WBC) count and WBC differentials. Meanwhile, blood serum were collected into slow sand filter (SST) tubes for biochemical evaluation that included measurements of total protein (TP), albumin, bilirubin, alkaline phosphatase (ALP), aspartate aminotransferase (AST) and alanine aminotransferase (ALT). Vital organs such as brain, liver, kidneys, heart, lungs and reproductive organs also were collected, sliced and stained with hematoxylin and eosin (H&E) at the end of the study for histopathological assessments.

**Specifications Table**

**Table:** 

Subject	Chemistry, toxicology

Specific subject area	Plant extract characterization, acute toxicity and subacute neurotoxicity profiling of flavanoid-enriched fraction (FEF) extracted from *Oroxylum indicum* on Sprague Dawley (SD) rats
Type of data	Table, image
How the data were acquired	FEF were extracted from the leaves of *O. indicum* using dual-solvent (petroleum ether and methanol) soxhlet extraction method, followed by fractionation enrichment process using Diaion HP20 resin column flushed with methanol as mobile phase. The obtained FEF was then subjected to liquid chromatography-mass spectroscopy (LC-MS) for detailed profiling. The LC system was equipped with an autosampler and Waters Acquity UPLC-HSS T3 column, 1.8 µm, 100 µm × 100 mm. The detection was performed through direct injection mode using Electron Spray Ionization (ESI) probe at positive-mode. The mobile phases were water + 0.1% formic acid (solvent A) and 90% acetonitrile in water + 0.1% formic acid (solvent B) at a flow rate of 500 µL/ min. The LC conditions were 1% of solvent B during 0-16 min, then increased from 1% to 35% of solvent B during 16–18 min and finally increased from 35% to 100% of solvent B during 18-20 min. Lastly, the system was turned to initial condition of 1% of solvent B at 20 min. The acquisition method was set in the range of minimum 50 dalton (m/z) to maximum 1000 dalton (m/z) with scanning rate of one spectrum per second. Water Vion IMS QTOF Mass Spectrometer was used for the MS analysis. The analysis was done using data-dependent automatic switching between MS and MS/MS acquisition modes.
	After confirming the content of FEF using LC-MS, SD rats were administered with the FEF based on the procedures set in Organisation for Economic Co-operation and Development (OECD) Guidelines 420 (Sighting and Acute Toxicity Studies) and Guidelines 424 (Subacute Neurotoxicity Study) in vivo. The behaviour, toxicity signs and neurological functions of treated rats were observed daily, with special attention given for the first 6 hours after the FEF administration. Body and organs weight were measured using standard measuring scale for rats. Hematological data were obtained using standard automatic blood cell analyzer (Pathlab, Malaysia). Biochemical data were analyzed using standard automatic biochemical analyzer (Pathlab, Malaysia). Histopathological assessments were scored using a specific binomial scoring methods developed by consultant histopathologist through validated methods published in previous studies.
Data format	Raw Analyzed
Description of data collection	Flavonoid compounds were extracted and enriched into FEF from the leaves of *O. indicum* and characterized using LC-MS system. The safety profile of obtained FEF was evaluated in SD rats using Sighting Study, Acute Toxicity Study and Subacute Neurotoxicity Study based on OECD Guidelines 420 and 424 in vivo. Mortality, behavioural and clinical toxicity signs of FEF-treated rats were recorded daily, body weight were measured weekly while vital organs were harvested, weighted and scored for determination of relative organs weight (ROW) and histopathological assessments at the end of the study period. Blood samples were collected using cardiac puncture into ethylenediaminetetraacetic acid (EDTA) tubes and serum separator tubes (SST), respectively, for evaluation of hematological and biochemical parameters by Pathlab, Malaysia.
Data source location	• Institution: Universiti Sains Malaysia, Health Campus • City/Town/Region: Kubang Kerian, Kelantan • Country: Malaysia • Latitude 6° 04’ 60.00” N and longitude 102° 16’ 60.00” E
Data accessibility	With the article

## Value of the Data

• *O. indicum* is a medicinal plant which contains potential flavonoid bioactive compounds known to exert beneficial effects for various ailments. However, the safety profiles of these compounds are not yet validated.
• This study provides valuable information of the flavonoids constituents in the flavonoid-enriched fraction (FEF) extracted from the leaves of *O. indicum* plant. The plant's chemical profile presented here could facilitate the advancement of high quality herbal medicinal products with standardized bioactive compounds.
• This study also presented the detailed data for pre-clinical acute oral toxicity and subacute neurotoxicity toxicity evaluation of the FEF by strictly following the OECD guidelines 420 and 424 in animal mode.
• The data presented would be beneficial for future development of the FEF for therapeutic usage in pre-clinical and clinical studies.
• The data also can be used as fundamental references for further studies of FEF in longer duration setting, its mechanism of toxicity and how it affects the animal metabolism.
• Researchers in toxicology and pharmacology could be benefited from these data.

## 1 Objective

This study was conducted to determine possible acute oral toxicity and subacute neurotoxicity effects that could be imposed by the FEF extracted from *O. indicum* plant with detailed evaluation on animal's behaviour, hematological and serum biochemical values, as well as histopathological assessments.

## 2 Data Description

The dataset in this article contains full LC-MS characterization profile of the FEF extracted from *O. indicum* as tabulated in Table 1. Furthermore, this article also presented dataset obtained from the Sighting Study, Acute Toxicity Study and Subacute Neurotoxicity Study based on OECD Guidelines 420 and 424. Animals in Sighting Study were single dosed with 5, 50, 300 and 2000 mg/kg FEF and observed for 14 days (*n* = 1/dosage/gender). Animals in Acute Toxicity Study were single dosed with the highest dosage which none of the animal showed sign of mortality in the Sighting Study (2000 mg/kg FEF) and were observed for 14 days (*n* = 5/dosage/gender). On the other hand, animals in Subacute Neurotoxicology Study were given repeated dose of FEF (50 mg/kg) and observed for 28 days (*n* = 5/dosage/gender). Throughout the Sighting Study, rat's survival and mortality were recorded for each dosage as tabulated in Table 2 and body weight were measured weekly, as tabulated in Table 3. For Acute Toxicity Study, animal's behaviour and clinical toxicity signs were recorded daily as tabulated in Table 4, while mean body weight of experimental rats were recorded weekly as tabulated in Table 5. At the end of the acute toxicity study, vital organs were harvested and blood samples were collected in two separate vacutainer for hematology and biochemical analyses, respectively. Table 6 tabulated the mean ROW, where the ratio of organs weight to body weight of animals were calculated, while Table 7 tabulated the histopatological scoring of vital organs. Tables 8 and 9 showed group mean hematology and biochemical data, respectively in male and female rats.

**Table 1:** Flavonoid compounds identified in FEF extracted from *O. indicum* plant.

Component name	Observed m/z	Observed RT (min)	Response
Genistein	271.0608	14.89	1139639
3′,4′,5,7-Tetrahydroxy-8-methoxyisoflavone	317.0657	12.7	991538
Oroxylin A	285.0755	12.81	413567
Baicalein	271.0599	11.3	272404
Chrysin	255.0661	16.6	251256
Tilianin	469.1106	12.77	231354
3′-Methoxydaidzein	285.0755	12.61	212277
7-Hydroxy-1-methoxy-2-methoxyxanthone	287.0552	11.19	207041
1,7-Dimethoxy-2,3-methylenedioxyxanthone	301.0712	14.4	153375
Galangin (Norizalpinin)	271.0604	13.93	135595
Procyanidin B2	601.1322	14.76	121540
5,7-Dihydroxy-2′-methoxyflavone	285.0755	12.8	89949
Scutellarein	287.0552	12.33	63509
Hinokiflavone	539.0978	16.61	52296
Procyanidin A2	599.1191	16.57	47018
Bavachromanol	363.1205	15.28	43364
Psoralenol	356.1495	10.79	31825
Gallocatechin(4α→8)-epicatechin	595.1448	12.17	27092
5,7,4′-Trimethoxy flavone	335.089	16.17	21169
Procyanidin B2 gallate	883.1686	12.63	20592
Bilobetin	553.1134	16.7	19399
Amentoflavone	539.0978	16.47	17335
1,6,7-Trihydroxy-2,3-dimethoxyxanthone	327.048	14.85	12867
Citflavanone	361.1037	16.33	12710
8-(Δ2-Isopentenyl)-5,7,3′,4′-tetrahydroxy-flavone	372.1439	8.63	12625
6-Aldehydo-isoophio-pogonone B	341.102	16.69	12453
Sophoraisoflavone A	370.1287	16.67	12387
6,7-Dimethoxy-2-(2-p-methoxyphenylethyl) chromone	363.1203	16.16	12290
Isoxanthohumol	377.138	16.62	12220
1,8-Dihydrxy-2,3,6-trimethoxyxanthone	319.0787	8.27	11744
Ombuine	331.0814	14.94	10237
Leucodelphinidin	323.0765	15.71	8474
3,8-Dihydroxy-4,10-dimethoxy-7-oxo-[2] benzopyrano[4,3-b] [1]benzopyran-7-(5H)-one	343.0814	16.48	8127
Cnidimol F	313.0709	15.11	8043
Flavanonol	241.0861	16.1	7841
dl-Umtatin	297.0756	10.79	7541
Kushenol I	455.2068	16.61	6510
Corylinal	283.0579	10.99	6127
1,5-Dihydrxy-2,3,4,7-tetramethoxyxanthone	371.0767	16.6	5893
Mururin A	471.0696	11.96	5869
(-)-Eriodictyol	311.053	15.46	5041
Divaricatol	357.097	16.04	4693
7-Methyltectorigenin	337.071	13.3	4653
Dichotomitin	359.0762	12.81	4572
Aloesone	255.0653	17.71	4146
Dehydrosilybin	503.0953	11.98	3966
Licochalcone A	361.1434	16.79	3829
5,7-Dihydroxy-6-methyl-3-(2′,4′-dihydroxybenzyl)chroman-4-one	339.0843	10.43	3543
3′,5′,β-Trihydroxy-3,4,4′,α-tetramethoxychalcone	399.108	11.39	3428
Noririsflorentin	395.0771	16.04	3394
Noranhyoicaritin	393.1337	15.89	3117
7-Hydroxy-5,3′,4′-trimethoxy flavone	351.0868	15.82	2952
Leucocyanidin	329.0659	13.48	2930
2,3-Dihydroiriegnin	380.1317	4.85	2800
(-)-epi-Afzelechin	297.0737	12.19	2534
Methyl ophiopogonone B	349.1051	12.77	2522
1,5-Dihydrxy-2,3,7-trimethoxyxanthone	341.0655	9.55	2474
3′-O-Methyltaxifolin	341.0655	9.55	2474
Dihydrokaempferol	311.0556	15.73	2343
Pinocembrin_1	257.0811	13.15	2321
Cinchonain Ⅰb	470.145	11.66	2303
Afrormosin	321.0761	15.96	2282
3-Methoxy-distylin	341.0655	12.83	2147
(3R)-4′-Methoxy-2′,3′,7-trihydroxy-isoflavanone	303.0843	15.08	2101
Chrysosplenetin B	397.0898	11.62	2005
7-Hydroxy-3,5,6,8,3′,4′-hexamethoxyflavone	441.1152	12.4	1897
6-Aldehydo-isoophio-pogonone A	372.108	13.37	1893
Silybin	500.1584	10.54	1827
Cirsilineol	367.0813	13.47	1689
(-)-Epicatechin	313.0707	10.05	1629
6-Hydroxykaempferol	303.0504	9.78	1603
Desmethylisoophiopogonone B	321.0762	14.06	1591
4,7,2′-Trihydroxy-4′-methoxyisoflavanol	311.0897	14.65	1524
5,8-Dihydroxy-2-(2-p-methoxyphenylethyl) chromone	335.089	16.17	1436
5-Hydroxy-4′,7-dimethoxyflavanone	323.0917	11.01	1353
5,7-Dihydroxy-4′-methoxyisoflavanone	309.0753	13.3	1287
Isosilybin	497.143	11.97	1271
Kushenol Ⅰ	431.1794	17.87	1106
Quercetagetin-6,7,3′,4′-tetramethyl ether	397.0907	14.39	1062
Kushenol M	531.2381	17.37	1024

**Table 2:** Sighting study – survival data (*n* = 1 for each group).

Groups	Sex	Dead/Total	Mortality rate (%)
Control	Male	0/1	0
	Female	0/1	0
5 mg/kg	Male	0/1	0
	Female	0/1	0
50 mg/kg	Male	0/1	0
	Female	0/1	0
300 mg/kg	Male	0/1	0
	Female	0/1	0
2000 mg/kg	Male	0/1	0
	Female	0/1	0

**Table 3:** Body weight of control and treated rats in sighting test (*n* = 1 for each group).

Groups	Body weight (g) at Day 0	Body weight (g) at Day 7	Body weight (g) at Day 14	Body weight changes between D0 and D7*	Body weight changes between D7 and D14*
Control (M)	301	309	335	+8g (+2.7%)	+26g (+8.4%)
Control (F)	191	200	215	+9g (+4.7%)	+15g (+7.5%)
5 mg/kg (M)	312	318	345	+6g (+1.9%)	+27g (+8.5%)
5 mg/kg (F)	184	200	210	+16g (+8.7%)	+10g (+5.0%)
50 mg/kg (M)	295	303	345	+8g (+2.7%)	+42g (+13.8%)
50 mg/kg (F)	225	235	240	+10g (+4.4%)	+15g (+6.4%)
300 mg/kg (M)	316	344	360	+28g (+8.8%)	+16g (+4.7%)
300 mg/kg (F)	219	229	235	+10g (+4.5%)	+6g (+2.6%)
2000 mg/kg (M)	270	295	320	+25g (+9.2%)	+25g (+8.5%)
2000 mg/kg (F)	203	212	230	+9g (+4.4%)	+18g (+8.5%)

M: male; F: female.

⁎ All weekly body weight changes recorded were within normal increment rate (< 20% deviation from initial weight), indicating absence of abnormality.

**Table 4:** Clinical toxicity signs and behaviour of treated rats in acute oral toxicity test (*n* = 5).

Clinical Toxicity Signs	0.5h	1h	2h	3h	4h	6h	D2	D3	D4	D5	D6	D7	D8	D9	D10	D11	D12	D13	D14
Mortality	A	A	A	A	A	A	A	A	A	A	A	A	A	A	A	A	A	A	A
Skin and fur changes	A	A	A	A	A	A	A	A	A	A	A	A	A	A	A	A	A	A	A
Bitting	A	A	A	A	A	A	A	A	A	A	A	A	A	A	A	A	A	A	A
Tremors	A	A	A	A	A	A	A	A	A	A	A	A	A	A	A	A	A	A	A
Diarrhea	A	A	A	A	A	A	A	A	A	A	A	A	A	A	A	A	A	A	A
Repetitive circling	A	A	A	A	A	A	A	A	A	A	A	A	A	A	A	A	A	A	A
Bizarre behavior (self-mutilation, walking backwards)	A	A	A	A	A	A	A	A	A	A	A	A	A	A	A	A	A	A	A
Mucous membrane secretion (eyes, nose)	A	A	A	A	A	A	A	A	A	A	A	A	A	A	A	A	A	A	A

**Behaviour**																			

Grooming	N	N	N	N	N	N	N	N	N	N	N	N	N	N	N	N	N	N	N
Vocalization	N	N	N	N	N	N	N	N	N	N	N	N	N	N	N	N	N	N	N
Bruxing	N	N	N	N	N	N	N	N	N	N	N	N	N	N	N	N	N	N	N
Salivation	N	N	N	N	N	N	N	N	N	N	N	N	N	N	N	N	N	N	N
Alertness	N	N	N	N	N	N	N	N	N	N	N	N	N	N	N	N	N	N	N

A: Absence; N: normal.

**Table 5:** Body weight of control and treated rats in acute oral toxicity test.

Groups	Body weight (g) at Day 0	Body weight (g) at Day 7	Body weight (g) at Day 14	Body weight changes between D0 and D7*	Body weight changes between D7 and D14*
Control (M)	286.80 ± 8.07	303.60 ± 5.81	310.60 ± 6.35	4 to 31 g (1.35 to 12.15%)	2 to 11 g (0.70 to 3.67%)
Treated (M)	313.60 ± 8.63	335.60 ± 9.09	354.00 ± 10.11	16 to 28 g (5.42 to 9.55%)	13 to 22 g (4.04 to 6.58%)
Control (F)	218.80 ± 2.65	213.40 ± 4.30	233.20 ± 1.39	-14 to 5 g (-6.51 to 2.28%)	8 to 30 g (3.57 to 14.56%)
Treated (F)	200.80 ± 3.13	212.40 ± 3.80	224.00 ± 2.00	9 to 14 g (4.68 to 6.67%)	6 to 18 g (2.78 to 8.95%)

M: male; F: female.

⁎ All the weekly body weight changes recorded were within normal increment range (< 20% of initial weight) indicating absence of abnormality.

**Table 6:** Relative organs weight (ROW) of control and treated rats in acute oral toxicity test.

Organ weight	Male (*n* = 5)			Female (*n* = 5)		
	Control (g)	Treated (g)	ROW changes (%)**	Control (g)	Treated (g)	ROW changes (%)**
Brain	***0.63 ± 0.04***	0.54 ± 0.03*	***-14.28***	***0.67 ± 0.02***	0.80 ± 0.02*	***19.40***
Heart	0.39 ± 0.02	0.38 ± 0.01	-2.56	0.41 ± 0.01	0.44 ± 0.01	7.31
Lung	***0.69 ± 0.04***	0.49 ± 0.01*	***-28.98***	***0.83 ± 0.07***	0.57 ± 0.01*	***-31.32***
Liver	3.97 ± 0.26	3.83 ± 0.19	-3.52	4.05 ± 0.13	4.18 ± 0.12	3.20
Kidney	0.73 ± 0.03	0.66 ± 0.03	-9.59	0.74 ± 0.02	0.70 ± 0.02	-5.40
Testis	***1.16 ± 0.04***	1.00 ± 0.04*	-13.79	-	-	-
Epidydemis	***0.16 ± 0.01***	0.13 ± 0.01*	-18.75	-	-	-
Ovary	-	-	***-***	***0.06 ± 0.01***	0.05 ± 0.01*	***-16.66***

⁎ showed significant difference at (p<0.05).

⁎⁎ All the ROW changes recorded were within normal increment range (< 20% of initial weight), indicating absence of abnormality.

**Table 7:** Histopathological scoring of vital organs of treated rats in acute oral toxicity test.

Observation in organs	Male (*n* = 5)		Female (*n* = 5)	
	Control	Treated	Control	Treated
***Brain***				
Neuronal degradation (Triangulated pyknotic nuclei)/Ischemic neuron	A	A	A	A
Inflammatory cells	N	N	N	N
Cell death /Gliosis	A	A	A	A
Blood vessel density	N	N	N	N
***Heart***				
Myocardial necrosis	A	A	A	A
Infiltration of inflammatory cells	N	N	N	N
Fibrosis	A	A	A	A
***Lung***				
Epithelial thickening (Hyperplasia)	A	A	A	A
Epithelial degeneration	A	A	A	A
Fibrosis	A	A	A	A
Edema	A	A	A	A
Inflammatory cells	N	N	N	N
Hemorrhage	A	A	A	A
Necrosis	A	A	A	A
***Liver***				
Necrosis	A	A	A	A
Changes in nuclear and cytoplasmic features	A	A	A	A
Inflammatory cells	N	N	N	N
Fibrosis	A	A	A	A
Steatosis	A	A	A	A
Balloning	A	A	A	A
***Kidney***				
Glomeruli abnormalities (Distorted/Diminished)	A	A	A	A
Tubules abnormalities (Dilation) /Atrophy	A	A	A	A
Edema	A	A	A	A
Necrosis	A	A	A	A
Inflammatory cells	N	N	N	N
***Testis***				
Distortion of testicular tissue architecture	A	A	-	-
Necrosis of tubular lining epithelium	A	A	-	-
Vacuolization	A	A	-	-
Interstitial tissue edema	A	A	-	-
Vascular dilation and congestion	A	A	-	-
Spermatogenesis	N	N	-	-
Sperm in epididymal lumen	N	N	-	-
***Ovary***				
Developing follicles	-	-	N	N
Congestion	-	-	A	A
Edema	-	-	A	A
Inflammatory cells	-	-	N	N
Hemorrhage	-	-	A	A

A: Absence; N: normal.

**Table 8:** Hematological values of control and treated rats in acute oral toxicity test.

Parameters	Male (*n* = 5)			Female (*n* = 5)		
	Control	Treated	Normal range	Control	Treated	Normal range
RBC (10^12^/L)	7.62 ± 0.29	7.72 ± 0.09	6.39 – 8.01	***7.04 ± 0.11***	7.90 ± 0.09*	6.39 – 8.01
Hb (g/dl)	14.64 ± 0.52	14.36 ± 0.22	10.4 – 16.5	***13.76 ± 0.26***	14.88 ± 0.23*	8.6 – 15.38
PCV (%)	41.60 ± 1.56	42.20 ± 0.66	18 - 48	***38.80 ± 0.66***	44.00 ± 0.54*	10 - 47
MCV (fl)	54.40 ± 0.24	54.40 ± 0.24	29.41 – 123.07	54.60 ± 0.24	54.40 ± 0.60	15.15 – 119.44
MCH (pg)	19.00 ± 0.00	18.40 ± 0.24	18.37 – 36.98	19.20 ± 0.20	18.60 ± 0.24	13.07 – 41.57
MCHC (g/dL)	***35.00 ± 0.00***	34.00 ± 0.00*	25.41 – 80.55	***35.40 ± 0.24***	34.20 ± 0.20*	21.16 - 95
Platelet (10^9^/L)	***504.00 ± 56.72***	650.60 ± 22.61*	423 - 1580	547.40 ± 46.61	632.00 ± 34.35	423 - 1580
WBC (10^9^/L)	6.78 ± 0.64	8.30 ± 0.60	3.00 – 9.22	6.00 ± 0.76	6.74 ± 0.45	2.58 – 7.34
WBC differential:						
Neutrophil (%) Eosinophil (%) Basophil (%) Lymphocytes (%) Monocytes (%)	***19.70 ± 0.80*** ***1.34 ± 0.34*** 2.42 ± 0.44 ***75.60 ± 1.33*** 0.94 ± 0.12	15.38 ± 0.60* 0.30 ± 0.06* 1.46 ± 0.18 81.80 ± 0.89* 0.86 ± 0.09	6.14-22.95% 0.54-3.39% 0.0-5.0% 69.68-86.89% 0.8-3.8%	***6.22 ± 1.72*** 0.36 ± 0.21 2.78 ± 0.81 ***88.74 ± 2.64*** ***1.90 ± 0.25***	14.38 ± 1.20* 0.60 ± 0.30 1.62 ± 0.32 82.52 ± 1.59* 0.88 ± 0.13*	4.27-18.48% 0.3-4.29% 0-3.5% 71.77-89.94% 0.8-3.9%

⁎ showed significant difference at (*p*<0.05). However, there was no measurement exceeded the normal range values complying with internationally accepted Clinical & Laboratory Standards Institute (CLSI) [1] and Quality Assurance and Standards for Veterinary Clinical Pathology (ASVCP) [2] guidelines, indicating absence of abnormality. RBC: red blood cells; Hb: haemoglobin; PCV: packed cell volume; MCV: mean corpuscular volume; MCH: mean corpuscular haemoglobin; MCHC: mean corpuscular haemoglobin concentration; WBC: white blood cells.

**Table 9:** Biochemical values of control and treated rats in acute oral toxicity test.

Parameters	Male (*n* = 5)			Female (*n* = 5)		
	Control	Treated	Normal range	Control	Treated	Normal range
Total protein (g/L)	52.60 ± 1.57	55.60 ± 0.68	51.10 – 64.55	55.40 ± 0.93	56.80 ± 1.39	51.10 – 64.55
Albumin (g/L)	32.60 ± 0.81	34.60 ± 0.40	26.88 – 38.55	***33.80 ± 0.73***	37.00 ± 1.00*	26.88 – 38.55
Bilirubin (UMOL/L)	1.00 ± 0.00	1.00 ± 0.00	1.00 – 2.00	0.60 ± 0.40	1.00 ± 0.00	1.00 – 2.00
ALP (IU/L)	223.00 ± 7.99	237.00 ± 7.44	160.8 – 838.3	152.60 ± 10.23	164.60 ± 8.99	150 – 724.2
AST (IU/L)	185.80 ± 41.34	115.00 ± 4.09	65 - 203	127.60 ± 24.98	84.40 ± 3.71	74 - 143
ALT (IU/L)	***72.40 ± 6.82***	59.60 ± 1.63*	19 – 100.7	59.60 ± 3.33	51.20 ± 1.28	14 – 84.4

⁎ showed significant difference at (*p*<0.05). However, there was no measurement exceeded the normal range values complying with internationally accepted CLSI [1] and ASVCP [2] guidelines, indicating absence of abnormality. ALP: alkaline phosphatase; AST: aspartate aminotransferase; ALT: alanine aminotransferase.

Meanwhile, for Subacute Neurotoxicity study, animal's behavior and clinical toxicity signs were recorded daily in Table 10, while mean body weight of experimental rats were recorded weekly as tabulated in Table 11. Mean hematological and biochemical data were tabulated Tables 12 and 13, respectively. Table 14 tabulated the mean ROW while Tables 15 and 16 tabulated the histopathological images and scoring, respectively.

**Table 10:** Clinical toxicity signs and behaviour of treated rats in subacute neurotoxicity test (*n* = 5).

Clinical Toxicity Signs	0.5h	1h	2h	3h	4h	6h	D2	D3	D4	D5	D6	D7	D8	D9	D10	D11	D12	D13	D14

Mortality	A	A	A	A	A	A	A	A	A	A	A	A	A	A	A	A	A	A	A
Skin and fur changes	A	A	A	A	A	A	A	A	A	A	A	A	A	A	A	A	A	A	A
Bitting	A	A	A	A	A	A	A	A	A	A	A	A	A	A	A	A	A	A	A
Tremors	A	A	A	A	A	A	A	A	A	A	A	A	A	A	A	A	A	A	A
Diarrhea	A	A	A	A	A	A	A	A	A	A	A	A	A	A	A	A	A	A	A
Repetitive circling	A	A	A	A	A	A	A	A	A	A	A	A	A	A	A	A	A	A	A
Bizarre behavior (self-mutilation, walking backwards)	A	A	A	A	A	A	A	A	A	A	A	A	A	A	A	A	A	A	A
Mucous membrane secretion (eyes, nose)	A	A	A	A	A	A	A	A	A	A	A	A	A	A	A	A	A	A	A

**Behaviour**																			

Grooming	N	N	N	N	N	N	N	N	N	N	N	N	N	N	N	N	N	N	N
Vocalization	N	N	N	N	N	N	N	N	N	N	N	N	N	N	N	N	N	N	N
Bruxing	N	N	N	N	N	N	N	N	N	N	N	N	N	N	N	N	N	N	N
Salivation	N	N	N	N	N	N	N	N	N	N	N	N	N	N	N	N	N	N	N
Alertness	N	N	N	N	N	N	N	N	N	N	N	N	N	N	N	N	N	N	N
Clinical Toxicity Signs						D15	D16	D17	D18	D19	D20	D21	D22	D23	D24	D25	D26	D27	D28

Mortality						A	A	A	A	A	A	A	A	A	A	A	A	A	A
Skin and fur changes						A	A	A	A	A	A	A	A	A	A	A	A	A	A
Bitting						A	A	A	A	A	A	A	A	A	A	A	A	A	A
Tremors						A	A	A	A	A	A	A	A	A	A	A	A	A	A
Diarrhea						A	A	A	A	A	A	A	A	A	A	A	A	A	A
Repetitive circling						A	A	A	A	A	A	A	A	A	A	A	A	A	A
Bizarre behavior (self-mutilation, walking backwards)						A	A	A	A	A	A	A	A	A	A	A	A	A	A
Mucous membrane secretion (eyes, nose)						A	A	A	A	A	A	A	A	A	A	A	A	A	A

**Behaviour**																			

Grooming						N	N	N	N	N	N	N	N	N	N	N	N	N	N
Vocalization						N	N	N	N	N	N	N	N	N	N	N	N	N	N
Bruxing						N	N	N	N	N	N	N	N	N	N	N	N	N	N
Salivation						N	N	N	N	N	N	N	N	N	N	N	N	N	N
Alertness						N	N	N	N	N	N	N	N	N	N	N	N	N	N

A: Absence; N: normal.

**Table 11:** Body weight of control and treated rats in subacute neurotoxicity test.

Groups (*n* = 5 each group)	Body weight (g) at Day 0	Body weight (g) at Day 7	Body weight (g) at Day 14	Body weight (g) at Day 21	Body weight (g) at Day 28	Body weight changes between D0 and D7*	Body weight changes between D7 and D14*	Body weight changes between D14 and D21*	Body weight changes between D21 and D28*
Control (M)	295.40 ± 26.60	312.40 ± 25.27	329.00 ± 25.43	347.20 ± 26.68	360.00 ± 27.78	-4 to 35 g (-1.18 to 15.69%)	12 to 21 g (4.87 to 8.13%)	9 to 24 g (2.56 to 8.60%)	7 to 23 g (2.60 to 6.38%)
Treated (M)	289.00 ± 7.02	308.20 ± 4.41	327.40 ± 5.17	343.00 ± 5.95	348.40 ± 7.34	6 to 26 g (-1.94 to 9.77%)	10 to 23 g (3.26 to 7.30%)	12 to 20 g (3.79 to 5.91%)	-13 to 13 g (-3.70 to 3.96%)
Control (F)	202.60 ± 8.90	208.00 ± 10.88	211.40 ± 9.67	216.60 ± 8.61	220.40 ± 9.08	-5 to 21 g (-2.79 to 9.63%)	-7 to 9 g (-2.92 to 4.07%)	-4 to 10 g (-1.72 to 4.97%)	2 to 8 g (0.83 to 3.50%)
Treated (F)	214.00 ± 7.39	216.40 ± 5.73	226.20 ± 7.73	230.00 ± 5.81	234.60 ± 8.38	-4 to 7 g (-1.67 to 3.62%)	3 to 16 g (1.50 to 6.80%)	-4 to 14 g (-1.76 to 6.89%)	-5 to 16 g (-2.18 to 6.37%)

M: male; F: female.

⁎ All the weekly body weight changes recorded were within normal increment range (< 20% of initial weight), indicating absence of abnormality.

**Table 12:** Hematological values of control and treated rats in subacute neurotoxicity study.

Parameters	Male (*n* = 5)			Female (*n* = 5)		
	Control	Treated	Normal range	Control	Treated	Normal range
RBC (10^12^/L)	8.58 ± 0.36	8.62 ± 0.17	6.39 – 9.65	8.24 ± 0.75	8.30 ± 0.47	6.39 – 9.03
Hb (g/dl)	15.40 ± 0.67	15.66 ± 0.33	10.4 – 16.5	14.80 ± 1.26	14.86 ± 0.85	8.6 – 15.38
PCV (%)	45.80 ± 1.77	45.60 ± 1.02	18 - 48	43.00 ± 3.96	44.40 ± 3.07	10 - 47
MCV (fl)	53.40 ± 0.60	53.00 ± 0.31	29.41 – 123.07	51.80 ± 0.20	53.00 ± 1.04	15.15 –119.44
MCH (pg)	18.00 ± 0.44	18.00 ± 0.00	17.10– 20.40	18.00 ± 0.31	17.80 ± 0.20	13.07 – 41.57
MCHC (g/dL)	33.80 ± 0.48	34.20 ± 0.20	25.41 – 80.55	34.60 ± 0.67	33.80 ± 0.96	21.16 - 95
Platelet (10^9^/L)	685.20 ± 103.98	586.20 ± 28.53	423 - 1580	460.00 ± 156.65	571.20 ± 103.45	423 - 1580
WBC (10^9^/L)	6.94 ± 0.96	4.88 ± 0.91	3.00 – 9.22	7.20 ± 0.10	3.62 ± 0.42	2.58 – 7.34
WBC differential:						
Neutrophil (%) Eosinophil (%) Basophil (%) Lymphocytes (%) Monocytes (%)	43.56 ± 16.98 0.20 ± 0.10 1.00 ± 0.75 52.86 ± 16.39 2.48 ± 1.14	12.90 ± 1.38 0.28 ± 0.08 2.70 ± 0.18 83.10 ± 1.30 1.02 ± 0.08	9.00-49.3%% 0.20-3.50% 0.0-5.0% 69.68-86.89% 0.8-3.8%	**20.92 ± 10.87** 0.88± 0.63 1.42 ± 0.84 75.38 ± 10.03 1.40 ± 0.68	3.62 ± 0.42* 0.76 ± 0.17 1.20 ± 0.35 80.20 ± 4.02 7.02 ± 5.99^a^	3.27-33.20% 0.3-4.29% 0-3.5% 71.77-89.94% 0.8-8.9%

⁎ showed significant difference at (*p*<0.05). However, there was no measurement exceeded the normal range values complying with internationally accepted CLSI [1] and ASVCP [2] guidelines, indicating absence of abnormality. RBC: red blood cells; Hb: haemoglobin; PCV: packed cell volume; MCV: mean corpuscular volume; MCH: mean corpuscular haemoglobin; MCHC: mean corpuscular haemoglobin concentration; WBC: white blood cells.

**Table 13:** Biochemical values of control and treated rats in subacute neurotoxicity study.

Parameters	Male (*n* = 5)			Female (*n* = 5)		
	Control	Treated	Normal range	Control	Treated	Normal range
Total protein (g/L)	55.80 ± 1.59	56.20 ± 1.49	51.10 – 64.55	58.20 ± 1.39	56.40 ± 1.74	51.10 – 64.55
Albumin (g/L)	33.60 ±0 .74	35.20 ± 0.80	26.88 – 38.55	34.20 ± 0.37	34.60 ± 0.97	26.88 – 38.55
Bilirubin (UMOL/L)	1.20 ± 0.20	1.20 ± 0.20	1.00 – 2.00	1.00 ± 0.00	1.00 ± 0.00	1.00 – 2.00
ALP (IU/L)	172.00 ± 7.58	181.00 ± 9.22	160.8 – 838.3	**172.40 ± 4.78**	139.20 ± 9.81*	130 – 724.2
AST (IU/L)	95.80 ± 3.56	97.00 ± 4.53	65 - 203	87.40 ± 4.96	94.20 ± 8.59	74 - 143
ALT (IU/L)	65.60 ± 2.87	58.80 ± 2.26	19 – 100.7	56.20 ± 1.68	53.00 ± 2.60	14 – 84.4

⁎ showed significant difference at (*p*<0.05). However, there was no measurement exceeded the normal range values complying with internationally accepted CLSI [1] and ASVCP [2] guidelines, indicating absence of abnormality. ALP: alkaline phosphatase; AST: aspartate aminotransferase; ALT: alanine aminotransferase.

**Table 14:** Relative organs weight of control and treated rats in subacute neurotoxicity study.

Organ weight	Male (*n* = 5)			Female (*n* = 5)		
	Control (g)	Treated (g)	ROW changes (%)*	Control (g)	Treated (g)	ROW changes (%)*
Brain	0.56 ± 0.05	0.59 ± 0.02	+5.35	0.84 ± 0.03	0.82 ± 0.02	-2.38
Heart	0.38 ± 0.02	0.37 ± 0.01	-2.63	0.40 ± 0.01	0.39 ± 0.02	+2.5
Lung	0.53 ± 0.01	0.49 ± 0.03	-7.54	0.77 ± 0.04	0.82 ± 0.09	+6.49
Liver	4.19 ± 0.12	4.26 ± 0.20	+1.67	3.50 ± 0.07	3.63 ± 0.24	+3.71
Kidney	0.67 ± 0.03	0.70 ± 0.03	+4.47	0.73 ± 0.02	0.74 ± 0.04	+1.36
Testis	0.99 ± 0.02	1.01 ± 0.03	+2.02	-	-	-
Epidydemis	0.15 ± 0.01	0.16 ± 0.01	+6.66	-	-	-
Ovary	-	-	*-*	0.03 ± 0.01	0.03 ± 0.01	0

⁎ All the ROW changes recorded were within normal increment range (< 20% of initial weight), indicating absence of abnormality.

**Table 15:** Histomorphologies of vital organs including brain, heart, lung, liver, kidney and reproductive organs (testis or ovary) of control and treated rats in subacute neurotoxicity study. There was no significant changes observed between the groups. (ne) neuron; (n) nucleus; (a) alveoli, (as) alveolar sacs; (cv) central vein, (hc) hepatocytes chords, (s) sinusoids (sp); (g) glomerulus, (t) tubules; mature sperm, (sg) spermatogenic cells, (isc) interstitial cells of Leydig; (f) follicles, (cl) corpus luteum.

**Table 16:** Histopathological scoring of internal organs of control and treated rats in subacute neurotoxicity study.

Observation in organs	Male (*n* = 5)		Female (*n* = 5)	
	Control	Treated	Control	Treated
***Brain***				
Neuronal degradation (triangulated pyknotic nuclei)	A	A	A	A
Ischemic neuron	A	A	A	A
Inflammatory cells	N	N	N	N
Cell death /Gliosis	A	A	A	A
Angiogenesis	N	N	N	N
***Heart***				
Myocardial necrosis	A	A	A	A
Infiltration of inflammatory cells	N	N	N	N
Fibrosis	A	A	A	A
***Lung***				
Epithelial thickening (Hyperplasia)	A	A	A	A
Epithelial degeneration	A	A	A	A
Fibrosis	A	A	A	A
Edema	A	A	A	A
Inflammatory cells	N	N	N	N
Hemorrhage	A	A	A	A
Necrosis	A	A	A	A
***Liver***				
Necrosis	A	A	A	A
Changes in nuclear and cytoplasmic features	A	A	A	A
Inflammatory cells	N	N	N	N
Fibrosis	A	A	A	A
Steatosis	A	A	A	A
Balloning	A	A	A	A
***Kidney***				
Glomeruli abnormalities (distorted/diminished)	A	A	A	A
Tubules abnormalities (dilation /atrophy)	A	A	A	A
Edema	A	A	A	A
Necrosis	A	A	A	A
Inflammatory cells	N	N	N	N
***Testis***				
Distortion of testicular tissue architecture	A	A	-	-
Necrosis of tubular lining epithelium	A	A	-	-
Vacuolization	A	A	-	-
Interstitial tissue edema	A	A	-	-
Vascular dilation and congestion	A	A	-	-
Spermatogenesis	N	N	-	-
Sperm in epididymal lumen	N	N	-	-
***Ovary***				
Developing follicles	-	-	N	N
Congestion	-	-	A	A
Edema	-	-	A	A
Inflammatory cells	-	-	N	N
Hemorrhage	-	-	A	A

A: Absence; N: normal.

## 3 Experimental Design, Materials and Methods

### 3.1 Standardized extraction of flavonoid-enriched fraction (FEF) from *O. indicum*

#### 3.1.1 Collection and identification of plant material

The fresh leaves of *O. Indicum* were collected from Kampung Pasir Parit, Pasir Mas, Kelantan, Malaysia (LGPS coordinate: latitude 5.905471, longitude 102.1884469). The taxonomical identification was authenticated and deposited in the herbarium of Universiti Sains Malaysia (USM) with the specimen voucher number USM Herbarium 11751.

#### 3.1.2 Crude extraction

The flavonoid compounds was extracted and enriched through a binary solvent procedure, as previously outlined [3]. Briefly, *O. indicum* leaves were washed, dried in oven at 50°C, and finely grounded with electrical grinder. A cellulose thimble containing 25 g of the powdered leaves was placed in a Soxhlet extractor and added with 300 ml of petroleum ether. The solvent was gently heated to the boiling temperature of 42-62°C until the solvent turned into dark green. The solvent was then discarded and 300 ml of fresh petroleum ether was added. The boiling procedure was repeated until the solvent color became clear and the solvent was discarded. Subsequently, the thimble was air dried to remove any residual petroleum ether before the next extraction step was applied using methanol. A volume of 500 ml methanol was added into the Soxhlet extractor and gently boiled at 62-65°C until the solvent turned into dark green color. The solvent was collected and 500 ml of fresh methanol was added. Once again, the boiling procedure was repeated until the solvent color turned clear and the solvent was collected. All of the collected solvent was concentrated using a rotary evaporator (Buchi AG, Flavil, Switzerland), weighted and stored at 4°C for further use.

#### 3.1.3 Fractionation to enrich flavonoid compounds

The fractionation protocols was performed as previously described with slight modifications [3]. Briefly, a Diaion HP20 resin column (Mitsubishi Chemical Corporation, Tokyo, Japan) was rinsed with 10% methanol as a mobile phase to establish column equilibrium. Subsequently, 5 g of crude extract powder obtained from the previous procedure was dissolved in 5 mL of methanol and then loaded onto the resin column. The column was eluted using a mixture of methanol and distilled water in a ratio of 9:1 to isolate the desired FEF. The collected FEF was dried using a rotary evaporator, weighted and stored at 4°C for further use.

### 3.2 Liquid chromatography-mass spectroscopy (LC-MS)

LC-MS assay was performed to validate the presence of flavonoids constituents in the FEF. The LC system was equipped with an autosampler and a Waters Acquity UPLC-HSS T3 column with particle size 1.8 µm, 100 µm × 100 mm. The detection was performed through direct injection mode using Electron Spray Ionization (ESI) probe at positive-mode. The mobile phases were water + 0.1% formic acid (solvent A) and 90% acetonitrile in water + 0.1% formic acid (solvent B) at a flow rate of 500 µL/ min. The LC conditions were 1% of solvent B during 0-16 min, then increased from 1% to 35% os solvent B during 16–18 min and finally increased from 35% to 100% of solvent B during 18-20 min. The system was then turned to initial condition at 1% of solvent B at 20 min. The acquisition method was set in the range of minimum 50 dalton (m/z) to maximum 1000 dalton (m/z) with scanning rate of one spectrum per second. The Water Vion IMS QTOF Mass spectrometer was used for the MS analysis using data-dependent automatic switching between MS and MS/MS acquisition modes.

### 3.3 Experimental animal and husbandry

The experiment was performed using healthy adult male and female SD rats (8-12 weeks old), with body weight ranging between 250-300 g. The animals were obtained from the Animal Research and Service Centre (ARASC), USM, Health Campus. The rats were maintained in the intramural facilities and fed with standard laboratory diets and water ad libitum. The animals were kept under laboratory conditions of temperature 22°C (± 3°C), with 12 h light and 12 h dark cycle. All animals were acclimatized for at least 5 days before the commencement of the studies. All the experimental procedures were reviewed and approved by the USM Institutional Care and Use Committee (IACUC) [Ethical number: USM/IACUC/2019/(120(1019)].

### 3.4 Study design

#### 3.4.1 Sighting study

Sighting study of FEF extracted from *O. indicum* was performed using the protocols described in OECD guidelines for Testing of Chemicals number 420 [4]. In the sighting study, a pre-screening of FEF dosages was conducted using minimal number of animals (1 male and 1 female rat per dosage) based on the requirements set in the guideline. Minimum number of rat per group was used to minimize unnecessary sacrifice of animals. After single administration of FEF starting from 5 mg/kg dosage by oral gavage, the animals were observed for their mortality and signs of toxicity such as ataxia, tremors, hyperactivity, salivation, diarrhea, sleep, lethargy and convulsion for up to 24 hours. In the event where the lowest fixed dose of 5 mg/kg FEF was seen to cause death or morbidity in any of the rats, FEF was assigned under GHS category 1 (highly toxic) and the study was terminated according to the OECD guidelines. On the other hand, if there was no mortality observed, subsequent dosage of 50 mg/kg of FEF was administered to another pair of male and female rats (*n* = 1 for each sex) and observed for another 24 hours. If still no mortality was observed, the sighting study was continued for 300 mg/kg and 2000 mg/kg of FEF, respectively, based on the OECD guidelines. One pair of male and female rats were given 10% DMSO and saline as a control. All animals were observed for 14 days. The highest dose which did not cause mortality after 14 days was selected as the sub-lethal dose (LD_50_) for subsequent acute toxicity test.

#### 3.4.2 Acute toxicity study

For acute toxicity study, animals were administered with the highest dose that did not cause mortality as tested in sighting study. In acute toxicity study, the animals were randomly assigned into two groups; control (*n* = 5/sex) and treated (*n* = 5/sex) groups and were observed for 14 days. Treatments were administered only once (single administration), and the animals were monitored for any signs of treatment-related toxicity for 14 days. Body weight, food and water intake were also monitored after dosing and on weekly basis throughout the experimental period.

#### 3.4.3 Subacute neurotoxicity study

Subacute neurotoxicity study of FEF extracted from *O. indicum* was conducted in accordance to OECD guidelines 424 for Testing of Chemicals [5]. A total of 20 SD rats (10 males and 10 females) were used in this study. The animals were randomly divided into 2 groups (5 males and 5 females/group). One group served as a control and was given diluted DMSO and saline (10 ml/kg b.wt). The other group was given 50 mg/kg b.wt of FEF via oral gavage for 28 successive days. All rats were observed for mortality, toxic symptoms and behavioral changes every day throughout the study duration. Neurological functional test, body weight, food and water intake were monitored after dosing and weekly basis throughout the experimental period.

### 3.5 Assessment parameters

#### 3.5.1 Blood hematological and biochemical assays

After the observation period, ketamine (100 mg/kg) and xylazine (5 mg/kg) b.wt was used to anaesthetize the rats. Blood samples were withdrawn through cardiac puncture into two separate tubes: ethylene diamine tetraacetic acid (EDTA) tubes for hematological assay and slow sand filter (SSF) tube for biochemical assay. A standard automatic blood cell analyser (Pathlab, Malaysia) was used to test for blood haematological parameters. The whole blood samples were analysed for red blood cell count (RBC), white blood cell count (WBC), WBC differentials (neutrophil, eosinophil, basophil, lymphocyte, and monocyte counts), haemoglobin (Hb), packed cell volume (PCV), mean corpuscular volume (MCV), mean corpuscular haemoglobin (MCH), mean corpuscular haemoglobin concentration (MCHC), and platelet count. The values of blood haematological parameters were compared to the standard reference values in healthy SD rats complying with internationally accepted Clinical & Laboratory Standards Institute (CLSI) [1] and Quality Assurance and Standards for Veterinary Clinical Pathology (ASVCP) [2] guidelines.

For serum biochemical indices, the blood was collected and centrifuged at 3500 rpm for 10 minutes for serum separation and subjected to biochemical analysis. The biochemical assay determination were for liver function profile that includes serum total protein (TP), albumin (ALB), total bilirubin (TBIL), alkaline phosphatase (ALP), alanine aminotransferase (ALT), and aspartate aminotransferase (AST). The assays were performed using standard automatic biochemical analyser (Pathlab, Malaysia). The values of serum biochemical indices were compared to the standard CLSI and ASVCP reference values in the same manner as described for the haematological assay.

#### 3.5.2 Relative organ weight (ROW)

At the end of the study, all the experimental rats were sacrificed and internal organs which included brain, heart, lungs, liver, kidneys and reproductive organs (testis and epidydemis or ovary) were carefully dissected and put into a Petri dish that contained 10% normal saline. The gross morphology of all selected internal organs were observed and recorded. This was to detect any signs of abnormalities or lesions on the organ. Then, each of the organ was dried with cotton wools and weighed on a sensitive balance. The parameter applied in this study was ROW (ratio of organ weight to body weight) to account for the differences of body weight in different animal. The weight of each organ was standardized to 100 g body weight of each rat at the day of sacrifice using the following formula:

$$ROW=\;\frac{Absolute\;organ\;weight\;\left(g\right)}{Body\;weight\;of\;rat\;on\;sacrifice\;day\;\left(g\right)}\;\times \;100$$

#### 3.5.3 Histopathological assessments

All the collected organs were fixed in 10% buffered formalin solution (pH 7.4) and preserved for the subsequent histopathological evaluation. The preserved organs were then processed using automated tissue processor and embedded in paraffin wax. Embedded tissue blocks were sliced into tissue slices of 3 µm in thickness using microtome. All tissue slices were stained with hematoxylin and eosin (H&E) dye and observed under light microscope to detect toxicity-related signs which included tissue degradation, lesions, inflammation, necrosis, apoptosis and leukocyte infiltration as described in previous studies [6], [7], [8], [9].

### 3.6 Statistical analysis

All statistical analysis was done using statistical package for the social sciences (SPSS version 27, USA). All data values were expressed in mean ± standard error of mean (SEM). Differences in all parameters for treated and control rats were analyzed using a One-way Analysis of Variance (ANOVA), followed by Tukey's test for multiple comparisons. Significant difference between the means of two groups was analyzed using T-test. All data with a p value of 0.05 or less (p<0.05) were considered as significant.

## Ethics Statements

The experimental procedures involving the care, handling and treatment of animals were reviewed and approved by the Universiti Sains Malaysia (USM) Institutional Care and Use Committee (IACUC) [Ethical number: USM/IACUC/2019/(120(1019)].

## Funding

This research was funded by Ministry of Higher Education Malaysia for the Fundamental Research Grant Scheme with Project Code: FRGS/1/2019/SKK08/USM/03/7.

## CRediT authorship contribution statement

**Farah Amna Othman:** Conceptualization, Methodology, Writing – original draft, Writing – review & editing. **Anani Aila Mat Zin:** Methodology. **Yusmazura Zakaria:** Methodology. **Nik Nur Hakimah Nik Salleh:** Methodology. **Suat Cheng Tan:** Conceptualization, Methodology, Writing – review & editing, Supervision, Project administration, Funding acquisition.

## Declaration of Competing Interest

The authors declare that they have no known competing financial interests or personal relationships that could have appeared to influence the work reported in this paper.

## Data Availability

Data set of Acute Oral Toxicity and Subacute Neurotoxicity Pre-clinical Risk Assessments on Sprague Dawley Rats Treated with Single Dose or Repeated Doses of Flavanoid-Enriched Fraction Extracted from (Original data) (Mendeley Data).
